# Diabetic cardiomyopathy: The role of microRNAs and long non-coding RNAs

**DOI:** 10.3389/fendo.2023.1124613

**Published:** 2023-03-07

**Authors:** Mirjana T. Macvanin, Zoran Gluvic, Jelena Radovanovic, Magbubah Essack, Xin Gao, Esma R. Isenovic

**Affiliations:** ^1^ Department of Radiobiology and Molecular Genetics, VINČA Institute of Nuclear Sciences - National Institute of the Republic of Serbia, University of Belgrade, Belgrade, Serbia; ^2^ University Clinical-Hospital Centre Zemun-Belgrade, Clinic of Internal Medicine, Department of Endocrinology and Diabetes, School of Medicine, University of Belgrade, Belgrade, Serbia; ^3^ King Abdullah University of Science and Technology (KAUST), Computer, Electrical, and Mathematical Sciences and Engineering (CEMSE) Division, Computational Bioscience Research Center (CBRC), Thuwal, Saudi Arabia

**Keywords:** diabetes, cardiomyopathy, microRNAs, long non-coding RNAs, therapeutic application

## Abstract

Diabetes mellitus (DM) is on the rise, necessitating the development of novel therapeutic and preventive strategies to mitigate the disease’s debilitating effects. Diabetic cardiomyopathy (DCMP) is among the leading causes of morbidity and mortality in diabetic patients globally. DCMP manifests as cardiomyocyte hypertrophy, apoptosis, and myocardial interstitial fibrosis before progressing to heart failure. Evidence suggests that non-coding RNAs, such as long non-coding RNAs (lncRNAs) and microRNAs (miRNAs), regulate diabetic cardiomyopathy-related processes such as insulin resistance, cardiomyocyte apoptosis and inflammation, emphasizing their heart-protective effects. This paper reviewed the literature data from animal and human studies on the non-trivial roles of miRNAs and lncRNAs in the context of DCMP in diabetes and demonstrated their future potential in DCMP treatment in diabetic patients.

## 1 Introduction

### 1.1 Diabetes mellitus and diabetes-related cardiomyopathy

Diabetes mellitus (DM) is a group of metabolic disorders characterized by chronic hyperglycemia and perturbed metabolism of carbohydrates, lipids, and proteins, resulting from defects in insulin secretion and action. An estimated 9.3% of the world population (463 million aged 20-79 years) is affected by DM, and this number is projected to reach 10.9% (700 million people) by 2045 (1). Macrovascular complications, such as coronary artery disease (CAD) and ischemic cardiomyopathy, are the leading causes of cardiac death in DM patients. In addition, DM raises the risk of heart failure (HF) and cardiac dysfunction unaided by other risk factors, such as CAD and hypertension (2). Also, microvascular disease and cardiac capillary rarefaction contribute to severe cardiovascular morbidity and mortality in DM patients (3, 4). Diabetes-related cardiomyopathy (DCMP) represents DM-induced morphofunctional cardiac abnormality after the presence of valvular, atherosclerotic, congenital, or hypertensive heart disease is excluded (5–7). Clinically, DCMP can be presented as two distinctive phenotypes, restrictive (heart failure with preserved ejection fraction, HFpEF) and dilated (heart failure with reduced ejection fraction, HFrEF) (8, 9). Transitioning from HFpEF to HFrEF is not mandatory (3, 9). In HFpEF and HFrEF, the presence of DM increases the risk of hospitalization for HF or even death (10, 11). However, the difficulty in identifying HF is due to the asymptomatic presentation in the early stages of DCMP (12). Furthermore, DCMP worsens DM patients’ prognoses and raises their chance for overt HF (13, 14).

The present challenges in the definitive diagnosis of DCMP are the absence of specific circulating or histological biomarkers of the disease (3, 15, 16) and insufficient guidance for managing patients suffering from both DM and HF (15, 17). Currently, DCMP diagnosis is most widely determined using echocardiography; to detect changes in the myocardium structure and function (9, 18–20). However, due to its economic costs, it is not well-suited for routine screening of DCMP. Thus, there is an urgent need to identify and develop novel blood-based biomarkers to identify patients with an increased risk of developing DCMP (18).

### 1.2 Role of non-coding RNAs in DCMP

Dysregulation of long non-coding RNA (lncRNA) and microRNA (miRNA) regulatory networks is emerging as an important mechanism in the pathophysiology of DCMP (3, 21–23). miRNAs are small, non-coding RNAs (ncRNA) that regulate the expression of numerous genes involved in physiological processes such as metabolism, apoptosis, differentiation, and cell proliferation. Increasing evidence points to miRNAs’ role in the regulation of pathophysiological alterations associated with DCMP, such as cardiac hypertrophy (24), myocardial fibrosis (25), oxidative stress (OS) and apoptosis (26), mitochondrial dysfunction (27), epigenetic modification (28), cardiac electrical remodeling (29). lncRNAs are long, non-translated transcripts with more than 200 nucleotides involved in regulating the activity and abundance of miRNAs through base-pairing interactions (22, 30). lncRNAs mediate numerous physiological processes, such as transcription regulation, RNA splicing, nuclear architecture and compartmentalization, and nuclear-cytoplasmic trafficking (31–34). Recent reports implicate the role of lncRNA in DM pathogenesis and associated cardiovascular complications, such as DCMP (35, 36).

In this review, we provide a systematic overview of DCMP pathogenesis and progression, focusing on the specific roles of miRNAs and lncRNAs in the pathophysiology of DCMP. Also, we discuss novel approaches based on the use of miRNAs and lncRNAs as targets for potential therapeutic interventions.

## 2 Diabetic cardiomyopathy: Pathogenesis, disease progression, and clinical presentation

### 2.1 DCMP pathogenesis

The pathogenesis of DCMP is based on joined metabolic conditions (hyperglycemia, hyperinsulinemia, and dyslipidemia) that promote OS, inflammation, the formation and deposition of advanced glycation end products (AGEs), damage and dysfunction of mitochondria, unbalanced Ca^2+^ homeostasis, endoplasmic reticulum stress (ERS), autonomic neuropathy, the renin-angiotensin system (RAS) activation, microvascular myocardial rarefaction, changes in gene regulation (microRNAs), and cardiomyocyte apoptosis (2, 5, 16, 37–39).

Both types of DM are characterized by decreased insulin signaling and changes in other signaling cascades, such as reduced AMPK and increased PKC and MAPK signaling, with resultant deleterious and maladaptive effects (3). DCMP’s clinical presentation may be preceded by myocardial structure changes and disturbed Ca^2+^ signaling and metabolism (2, 4, 7, 22). The myocardial structure changes, i.e., myocardial fibrosis, are favoured by increased collagen deposition and variations in extracellular matrix (ECM) protein structure (40). The imbalance between profibrotic factors, such as connective tissue growth factor and transforming growth factor β1, and the inactivity of the ECM-degrading enzyme metalloproteinase can lead to ECM accumulation (41, 42). Among numerous mechanisms that favour DM-induced cardiac fibrosis, the intriguing one is the endothelial-to-mesenchymal transition (EndMT). EndMT is known to be promoted by hyperglycemic conditions, and it evolves gradually, acquiring a fibroblastic phenotype while simultaneously losing the original phenotype of the endothelial cells (ECs). This phenotypic change is accompanied by a progressive decline in EC activity and the cells’ mesenchymal characteristics, such as increased ECM protein production, becoming more pronounced. In injured tissue, the EndMT-derived cells act as immature fibroblasts and promote the fibrosis process (43).

Cardiomyocytes with abnormal metabolism are susceptible to increased free fatty acid (FFA) uptake and oxidation. Increased lipids may promote cardiomyocyte death induced by lipotoxicity due to limited FFA oxidation (3, 44). In addition, reactive oxygen species (ROS) and reactive nitrogen species (RNS) are produced more frequently as a result of increased intracellular fatty acid content and mitochondrial malfunction, which in turn increases OS and ERS and inhibits autophagy (20, 45, 46). The interaction of these effects causes ECM remodeling and fibrosis, along with cardiomyocyte loss, cardiac enlargement, and inflammation (47). Heart stiffness, poor cardiac relaxation, and diastolic dysfunction are early signs of DCMP caused by pathophysiological anomalies (46). In addition, accumulated lipids in ECs may decrease nitric oxide (NO) bioavailability, promoting endothelial dysfunction and accelerating atherosclerosis (9).

Diastolic or systolic dysfunction is encouraged by left ventricular (LV) hypertrophy and perivascular and interstitial cardiac fibrosis (3, 48). On echocardiograms, LV hypertrophy presents increased thickness in the posterior and septal walls (49). Myocyte hypertrophy, thickening of the myocardial capillary basement membrane, and increased interstitial and perivascular fibrosis are confounding factors contributing to the development of LV hypertrophy (50, 51).

### 2.2 Natural course and diagnostic management of DCMP

DCMP occurs in approximately 12% of patients with DM (52). The prevalence of HF varies between 19 and 26% in both types of DM (3, 4). The link between type 2 DM (DMT2) and HF is bidirectional: HF is highly prevalent in DMT2 patients, and HF increases the risk of DMT2 (3, 53, 54). Sometimes, HF is the first cardiovascular presentation in patients with DMT2 (9, 55). In DM patients, the risk of a negative HF outcome is greater (56). The risks of HF in diabetic patients are in close relation to the quality of retrograde glycemic control, as the patients with type 1 (DMT1) and DMT2 have a 30% and 8% increase in HF risk for each 1% increase in glycated haemoglobin (HbA1c) level, respectively (3, 57).

DCMP’s natural course is determined by phenotype. In DCMP with HFpEF phenotype, the LV is hypertrophied, stiff, and of normal size. In DCMP with HFrEF phenotype, the LV is dilated with reduced ejection fraction (9, 58). In humans, diastolic dysfunction almost always precedes the development of systolic dysfunction (59, 60). Metabolic abnormalities in DMT2 predispose to the development of HFpEF DCMP, while the autoimmune abnormalities in DMT1 favour HFrEF DCMP (9).

At present, echocardiography represents an indicative diagnostic tool for assessing a patient with suspected DCMP (16, 61). The first echocardiographic signs of DCMP are LV diastolic dysfunction and mechanical changes leading to HFpEF and, ultimately, HFrEF (19). In the early stage or the restrictive HFpEF form, the echo findings show normal LV diameters and volumes with concentric hypertrophy, preserved systolic function (EF ≥50%), and indications of diastolic dysfunction (9). Systolic dysfunction is a later manifestation, sometimes misdiagnosed using standard two-dimensional echocardiography (2). Rarely, T1 cardiac MRI mapping is an initial diagnostic procedure in detecting DCMP, as myocardial ECM in DM patients and non-DM controls exhibit significant differences (62). In addition, the increased levels of natriuretic peptide, inflammatory markers, and cardiac fibrosis markers are linked to diastolic dysfunction in DCMP (16, 63, 64). In the advanced stages of DCMP, or the dilated/HFrEF form, systolic dysfunction (ejection fraction <50%) occurs and an increase in LV volume (9).

Continuous inflammatory stimulation appears to be one of the most critical factors of DM pathogenesis (65). In the acute phase of inflammation, cytokines and acute-phase proteins (APPs) mitigate the effects of transient inflammatory processes (66, 67). However, prolonged inflammation results in a chronic condition where immune response leads to tissue damage contributing to the pathogenesis of many diseases, including atherosclerosis, cardiomyopathy, and DM (68). Nevertheless, diagnostics of DCMP based on measurements of circulating markers of inflammation, such as complement compounds, C-reactive protein (CRP) and alpha-macroglobulin (α2M), and amyloid A and P, is not sufficiently reliable, thus requiring identification of more specific biomarkers that enable early detection of DCMP (18, 68).

#### 2.2.1 miRNAs and lncRNAs as potential biomarkers for DCMP

Circulating miRNAs and lncRNAs have been recently proposed as novel type of biomarkers for the diagnosis of cardiovascular disease (CVD), primarily due to their involvement in epigenetic mechanisms that underpin the progression of cardiomyopathies (69–71). Crucial attributes that support their use as potential biomarkers are their abundance and long-term stability in various body fluids (72, 73). In recent years, mounting evidence based on observation of expression patterns of various miRNAs and lncRNAs using high-throughput sequencing methodologies points at their use as reliable and reproducible prognostic and diagnostic biomarkers for various diseases, including DCMP. For instance, numerous clinical and experimental studies proposed various circulating miRNAs as biomarkers for diabetes prognosis (74–76) and the diagnosis of myocardial infarction, cardiac hypertrophy, and myocardial fibrosis (77–80). Similarly, several lncRNAs have been reported to play a crucial role in cardiovascular complications of diabetes and were implicated as potential biomarkers for DCMP (23, 81–83). In the following sections of this review, we provide a more detailed overview of specific miRNAs and lncRNAs emerging as novel, reliable DCMP biomarkers, thus representing valuable addition to existing prognostic and diagnostic tools for DCMP.

### 2.4 Treatment of DCMP

Stringent control of DM and the treatment of HFpEF or HFrEF is the cornerstone of DCMP management. DCMP is not a rare cardiovascular complication of DM (16). Using tissue Doppler strain analysis and measurements of peak systolic velocity, almost every fifth patient with DM was diagnosed with systolic dysfunction after excluding CAD or hypertension (49). Novel oral agents currently used in DM management (i.e., sodium-glucose cotransporter 2 (SGLT2) inhibitors, glucagon-like peptide 1 receptor agonists (GLP1-RAs)) enable a reduction in hospitalization rates for HF in DM patients independently of the presence of HF at baseline (84, 85). SGLT2 inhibitors exert antioxidative, antiapoptotic, and anti-inflammatory effects and decelerate atherosclerosis (86).

## 3 miRNAs in diabetic cardiomyopathy

### 3.1 General characteristics of miRNA

miRNAs represent small (17-25 nucleotides), single-stranded non-coding RNA molecules that regulate gene expression (87). Theoretically, a single miRNA could bind to over 1000 target mRNAs, and various miRNAs could regulate the expression of the same target transcript (88, 89). Since each miRNA may target several mRNAs, it has been estimated that miRNAs may regulate the expression of up to 60% of protein-coding genes in humans (90). Until 2019, the miRBase database (miRBase Release 22.1, https://www.mirbase.org/) reported entries of 38 589 miRNAs in 271 species, including 2654 mature human miRNAs (91). Increasing evidence supports the significant roles of miRNAs in regulating the mechanisms responsible for the pathophysiology of numerous diseases, including cardiovascular diseases, obesity, different types of cancer, and diabetes (73, 92–98).

miRNAs biogenesis is a multistep process that starts with primary miRNA (pri-miRNA) transcription by RNA polymerases II and III in the nucleus, which is subsequently processed by the nuclear endoribonuclease DROSHA or by components of the splicing machinery (99) to approximately 70 nucleotides long precursor (pre-miRNA) molecules that are exported to the cytoplasm by exportin 5 and Ran-GTPase. Additional processing by type III endoribonuclease DICER associated with RNA-binding proteins yields mature double-stranded miRNAs. The guide strand of mature miRNAs associates with Argonaute (AGO) proteins or chaperones HSC70/HSP90 to form the minimal miRNA-induced silencing complex (miRISC) that binds to the target mRNA’s complementary sequences called miRNA response elements (MREs). MiRNAs mainly interact with the target mRNAs’ 3′ untranslated regions (UTR) to induce translational repression and mRNA deadenylation (100–102), but interactions of miRNAs with 5′ UTR, gene promoters, and coding sequences have also been observed (103). It is generally assumed that the interaction of miRNAs with coding regions and 5′ UTR silence gene expression (104, 105), while binding of miRNAs to promoter regions can trigger transcription (106).

### 3.2 Role of miRNAs in cardiomyocyte hypertrophy and myocardial apoptosis

#### 3.2.1 miRNAs expression and glycemic status in DCMP

The involvement of miRNAs in DM-associated pathophysiological processes in the myocardium is supported by findings that more than 300 different miRNAs have altered expression in DCMP (23). Expression of numerous miRNAs influences cardiomyocyte survival by modulating response to OS and inflammation (107, 108). In addition, levels of different miRNAs correlate with glycemic status, i.e., ‘miRNAs’ synthesis is influenced by high glucose levels (109, 110). This effect is likely mediated by endonucleases DROSHA and DICER, which is supported by a recent study by Lam et al. demonstrating that high glucose reduces DROSHA protein levels (111). Also, Chavali et al. measured the levels of pro-inflammatory tumour necrosis factor-alpha (TNFα), anti-inflammatory interleukin-10 (IL-10), DICER, and miRNAs in hearts of Akita, a genetic mice model for diabetes, and C57BL/6J (WT). The study reported increased mRNA and DICER levels in Akita’s hearts compared to the wild-type ones (112). Subsequent miRNA array analysis showed significant downregulation of several miRNAs, including miR-872, miR-744, miR-542-3p, miR-500, miR-499, miR-494, miR-455, miR-451, miR-450, miR-433, miR-384-3p, miR-345-3p, miR-338, miR-148, miR-142-3p, miR-130, and let-7a. Only one miRNA, miR-295, was found to be upregulated (112), which is in agreement with data from Baseler et al. showing increased levels of miR-295 in DMT1 myocardium (113).

The development of DCMP depends on several mechanisms mediated by mitogen-activated protein kinase (MAPK)-mediated signaling pathways, including inflammation, OS, and extracellular fibrosis. Of particular importance is p38 MAPK which is activated during cardiomyocyte hypertrophy, apoptosis, inflammation, OS, and conditions of metabolic abnormalities (114–117). Increasing evidence demonstrates that p38 MAPK expression is perturbed in the heart in diabetic conditions and that inhibiting p38 MAPK activation with its inhibitor atorvastatin or in a transgenic animal model prevents DCMP development (118, 119). Furthermore, dysregulated miRNAs in the hearts of diabetic mice appear to be primarily associated with the MAPK signaling pathway. For instance, *in vitro* inhibition of p38 MAPK decreases miR-373 expression, and miR-373 was shown to be significantly downregulated in the cardiac tissue of diabetic mice. Additionally, experiments with rat cardiomyocytes exposed to high glucose *in vitro* and transfected by miR-373 show miR-373 overexpression accompanied by hypertrophy and decreased transcription factor MEF2C, suggesting that the *MEF2C* gene is the target of miR-373. Thus, p38 MAPK/miR-373/MEF2C was proposed as a regulatory pathway in glucose-dependent cardiomyocyte hypertrophy (**Table 1**) (120).

**Table 1:** The roles of miRNAs and lncRNAs in DCMP.

ncRNA	Expression	Target	Signaling pathway	Pathophysiological mechanism	Experimental model	References
miRNAs						
mir-373	↓	*MEF2C*	P38 MAPK	Cardiomyocyte hypertrophy	STZ-induced diabetes mouse model, neonatal rat myocytes	(118–120)
mir-30c	↓	*PGC-1β*, *Cdc42, Pak1*	PPARα, p53-p21	Cardiomyocyte hypertrophy OS	STZ-induced diabetes mouse model, neonatal rat cardiomyocytes	(121, 122)
mir-203	↓	*PIK3CA*	PI3KT/Akt	Cardiomyocyte hypertrophy OS Fibrosis Apoptosis	STZ-induced diabetes mouse model	(123)
mir-1	↓	*Junctin*	Ryanodine receptor calcium release channels	OS	STZ-induced diabetes mouse and rat models	(124–128)
miR-503	↑	*Nrf2*	Nrf	OS Apoptosis	STZ-induced diabetes Wistar rats, rat primary cardiomyocytes	(129)
miR-22	↓	*Sirt1*	Sirt1	OS Apoptosis	STZ-induced diabetes mouse model, embryonic cardiac myoblast cellline (H9c2 cells)	(129)
mir-21	↑	*LAZ3, PDCD4*	PPARα, Nrf2, NF-κB	OS Inflammation Apoptosis	STZ-induced diabetes mouse model, neonatal rat myocytes	(114, 130–132)
miR-150-5p	↑	*Smad7*	NF‐κB, TGF‐β1	Inflammation Fibrosis	HG-induced diabetes model, rat cardiac fibroblasts	(78, 133–137)
lncRNAs						
KCNQ1OT1	↑	miR-214-3p, *CASP1*	TGF-β1/Smad	Inflammation, fibrosis	STZ-induced diabetes mouse model, human blood serum from diabetic patients	(138, 139)
H19	↓	miR-675, *VDAC1, DIRAS3*	mTOR	Inflammation Apoptosis	STZ-induced diabetes rat model, neonatal rat myocytes	(134, 140)
MALAT1	↑	miR-26a, *HMGB1, SAA3*	TLR4/NF-κB	Inflammation Apoptosis	Human adult ventricular cardiomyocytes (AC16 cell line), STZ-induced diabetes mouse model	(140–142)
NONRATT007560.2	↑	miR-208a	TNFα	Inflammation Apoptosis OS	HG-induced diabetes model, rat cardiomyocytes	(143–145)
HOTAIR	↓	mir-34*, Sirt1*	PI3K/Akt	Inflammation Apoptosis OS Fibrosis	STZ-induced diabetes mouse model, rat cardiomyocytes	(146, 147)
ANRIL	↑	*HBEGF, CDH5*	TNFα	Inflammation Apoptosis OS Fibrosis	STZ-induced diabetes rat model	(148, 149)

↑/↓indicates the up/down-regulation of ncRNA expression.

ANRIL, Antisense Noncoding RNA gene at the INK4 locus; CASP1, caspase-1; Cdc42, Cell Division Cycle 42; CDH5, cadherin 5; DIRAS3, DIRAS Family GTPase 3; H19, H19 imprinted maternally expressed transcript; HBEGF, Heparin-Binding EGF-like Growth Factor; HG-high glucose; HMGB1, High Mobility Group Box 1; HOTAIR, HOX Transcript Antisense Intergenic RNA; LAZ3, Lymphoma-associated zinc finger 3; MALAT1, Metastasis Associated Lung Adenocarcinoma Transcript 1; MAPK, Mitogen-Activated Protein Kinases; MEF2C, Myocyte Enhancer Factor 2C; mTOR, Mammalian Target of Rapamycin; NF-κB, Nuclear Factor kappa-light-chain-enhancer of activated B cells; Nrf2, Nuclear factor erythroid 2–related factor 2; OS, Oxidative stress Pak1, P21 Activated Kinase 1; PGC-1β, Peroxisome Proliferator-activated receptor-γ co-activator 1 beta; PDCD4, programmed cell death 4 gene; PI3KT/Akt, Phosphatidylinositol 3-kinase/protein kinase B; PIK3CA, Phosphatidylinositol-4,5-bisphosphate 3-kinase catalytic subunit alpha; PPARα, Peroxisome Proliferator-Activated Receptor alpha; SAA3, Serum Amyloid A3; Sirt, Sirtuin; STZ, Streptozotocin; TGF‐β1, Transforming Growth Factor β; TLR4, Toll-Like Receptor 4; TNFα, Tumour Necrosis Factor alpha; VDAC1, Voltage-Dependent Anion Channel 1.

**Figure 1:**
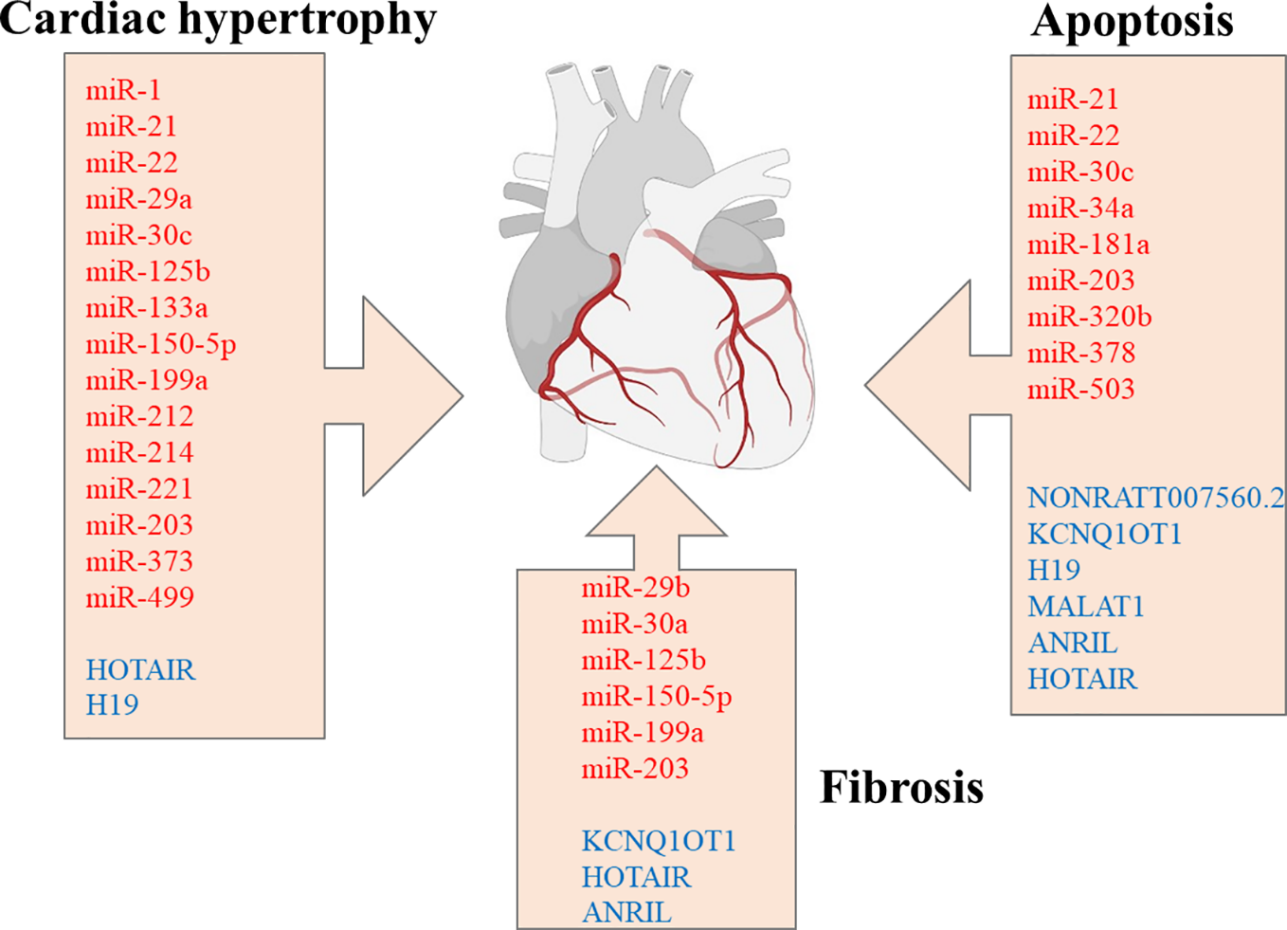
miRNAs and lncRNAs are implicated in regulating cardiac hypertrophy, apoptosis, and fibrosis. miRNAs are marked in red, whereas lncRNAs are marked in blue color. miRNAs, microRNAs; lncRNA, long non-coding RNAs. Created with Biorender.com.

LV miRNA profiling, from streptozotocin-induced diabetic mice, with or without intensive glycaemic control by slow-release insulin implants, demonstrated differential expression of 316 miRNAs. Among the dysregulated miRNAs, downregulation of miR-1 and upregulation of miR-19b, miR-27a, miR-34a, miR-125b, miR-146a, miR-155, miR-210, miR-221 was significant (127). Surprisingly, most dysregulated miRNAs’ expression remained significantly altered after normalization of the glucose levels in diabetic mice. Ingenuity Pathway bioinformatic analysis shows the dysregulated miRNAs were involved in physiological processes such as hypertrophic growth (miR-212, miR-221, miR-125b, miR-29a, miR-214, miR-133a, miR-199a, miR-150, miR-1), apoptosis (miR-320b, miR-378, miR-34a), fibrosis (miR-125b, miR-150, miR-199a, miR-29b, miR30a) (**Figure 1**), OS (miR-155, miR-27a, miR-125b, miR-19b, miR-221, miR-210, miR-146a, miR-34a), autophagy (miR-133a, miR-221, miR-212, miR30a), and heart failure (miR-423, miR-499, miR-199a). Of particular importance is a set of downregulated miRNAs associated with OS. For instance, miR-221, upregulated in the diabetic myocardium, was suggested to have a key role in the progression of diabetic myocardial damage after restoring normoglycemia, whereas miR-34a may be responsible for cardiac ageing in DM (127). Normalization of glucose levels failed to restore the downregulated miR-1, whose dysregulation is associated with arrhythmias, myocardial hypertrophy, myocardial infarction, and cell reprogramming (126–128). Mir-1 directly targets junctin, a component of the ryanodine receptor Ca^2 +^ release channel complex, and abolishes its expression (**Table 1**) (126). In high glucose conditions, decreased levels of miR-1 result in an elevated expression of junctin, which is associated with perturbed Ca^2 +^ handling, consequently causing arrhythmia and cardiac hypertrophy (124, 125).

The study by Constatino et al. suggests that failure to restore normal levels of dysregulated miRNAs in diabetic myocardium upon achieving normoglycemia may explain the progression of diabetic cardiovascular complications. It confirms the concept of metabolic memory, which was previously proposed but is insufficiently documented at the molecular level (150). Inhibition of OS-related miRNAs (miR-221, miR-210, miR-155, miR-146a, miR-125b, miR-34a, miR-27a, miR-19b) identified in this study may serve as a potential novel therapeutic strategy, leading to the amelioration of adverse effects of hyperglycaemic memory in diabetic myocardium.

#### 3.2.2 miRNAs involvement in PPAR and Nrf signaling

Sufficient evidence supports the role of miRNAs in modulating cell response to OS, which plays a vital role in the progression of diabetic myocardial dysfunction (122, 150). The activation of Nrf2, a transcriptional factor acting as an essential regulator of OS genes, is increased in DM models due to excessive ROS accumulation (151). Also, transcriptional factors activated by fatty acids, such as PPARα, exhibit anti-inflammatory activity by decreasing the expression of pro-inflammatory genes (152). Several studies report synergistic action of Nrf2 and PPARα signaling pathways (153, 154), where PPARα pathway activation leads to Nrf2 activation *via* PGC-1α (155). Yin et al. reported that miR-30c has a protective role in diabetic cardiomyopathy *via* PPARα (122). miR-30c levels were downregulated in the T2D1 diabetic model leading to an increased expression of PGC-1β, a direct target of mir-30c, resulting in metabolic disturbances, cardiac lipotoxicity, and augmented ROS production (122). The overexpression of miR-30c reduced myocardial lipid accumulation and excessive ROS production, improved glucose utilization, and attenuated cardiomyocyte apoptosis and cardiac dysfunction *in vitro* and db/db mice (122). Another study reported that miR-30c overexpression in rat cardiomyocytes under high-glucose treatment was accompanied by the downregulation of prohypertrophic genes *Cdc42* and *Pak1*, leading to cardiomyocyte hypertrophy attenuation (156). MiR-30c is also linked to the p53-p21 pathway involved in cardiomyocyte hypertrophy and apoptosis in DCM, and its effects may be amplified by miR-181a (121). Cardiomyocyte miR-30c overexpression in the DCM model led to an increased LV ejection fraction and reduced LV mass compared to controls (135). The attenuation of cardiac dysfunction by miR-30c overexpression suggests that miR-30c may be a potential therapeutic target for DCM treatment (122).

Regulation of PPARα and Nrf2 activation is also associated with miR-21 and *LAZ3* gene, a transcriptional repressor that interferes with NF-κB signaling, thus regulating inflammation (132). *LAZ3* expression is decreased in rat cardiomyocytes and diabetic mouse hearts (Gao, 157). *LAZ3* silencing upregulates expression of miR-21, which targets PPARα, consequently downregulating PPARα and Nrf2 signaling pathway and promoting an inadequate response to the OS. Gao et al. proposed that treatments based on miR-21 inhibitors may positively affect DCMP management (Gao, 157). However, the results of other studies conflict with this conclusion and suggest that overexpression of miR-21 may be a promising therapeutic approach for the treatment of DCMP (131). It was found that miR-21 overexpression protects against ROS-induced damage in cardiac myocytes *via* another target gene, *PDCD4*, and in cardiac nonmyocyte cells such as fibroblasts, diminished miR-21 expression reduces abnormal heart remodeling (130, 158). Increased levels of cardiac OS biomarkers observed in cardiomyocytes of diabetic mice were significantly decreased upon miR-21 treatment and phospho-Akt and phospho-endothelial Nitric Oxide Synthase (eNOS) overexpression suggesting that miR-21 attenuates cardiac hypertrophy by reducing ROS levels and increasing available NO (131). It appears that miR-21 may have different roles in different cell types and pathophysiological conditions, requiring further studies on human subjects to explain the reported contradictory findings.

Perturbed levels of several other miRNAs in the diabetic myocardium, such as upregulation of miR-503 and downregulation of miR-22, were observed *in vivo* and *in vitro* DCMP models (**Table 1**). Those miRNAs are suggested to impair the ability of Nrf2 to prevent the adverse effects of excessive ROS accumulation observed in DM. miR-503 upregulation is associated with Nrf2 activation that can be further enhanced through the phase II enzyme inducer CPDT, an enzyme complex with a protective role against OS by promoting antioxidative ‘enzymes’ expression (129). Decreased expression of miR-503, accompanied by increased Nrf2 levels and reduced development of cardiomyopathy, was observed in diabetic rats treated with CPTD compared to a control group (129). In the case of miR-22, whose levels were decreased in the myocardium of streptozotocin-induced diabetic mice, it was reported to target 3’- untranslated repeats of *Sirt1* and upregulate its expression (**Table 1**) (159). In a diabetic animal model, overexpression of miR-22 was associated with decreased ROS levels, elevated SOD, and amelioration of blood glucose levels, LV end-diastolic pressure, ejection fraction, and ‘cardiomyocytes’ apoptosis (129).

#### 3.2.3 miRNAs-mediated modulation of PI3K/Akt and NF‐κB signaling pathways

PI3K/Akt signaling pathway has a crucial role in the pathogenesis of insulin resistance and DCMP development, regulating multiple physiological processes, such as cell growth, the proliferation of cardiomyocytes, and apoptosis (160). PI3KT/Akt regulates the nuclear factor-κB (NF‐κB) transcriptional activity that regulates cellular activities related to immune responses and inflammation (161). Also, PI3KT/Akt is involved in platelet activation, which is associated with TGF-β1 release that promotes atrial fibrosis in cell culture and ventricular fibrosis in a mouse model (162, 163). It was reported that upregulation of miR-203 inhibits activation of the PI3KT/Akt pathway by targeting *PIK3CA* and is associated with reduced cardiac hypertrophy, myocardial apoptosis, fibrosis (**Figure 1**), and levels of ROS in myocardial tissues of diabetic mice (**Table 1**) (123). Another study reported that NF‐κB activity and IL‐1β production are significantly increased in cardiac fibroblasts under high glucose conditions and are accompanied by upregulation of miR-150-5p, which negatively regulates *Smad7* expression at the post-transcriptional level (137). Since *Smad7* was shown to suppress TGF‐β1 signaling (164), miR-150-5p inhibition attenuates ‘cardiomyocytes’ fibrosis and inflammation mediated by NF‐κB and TGF‐β1/Smad pathways. In addition, miR‐150‐5p involvement in the inflammatory cytokine production, the development of T and B lymphocytes, and vascular remodeling and fibrosis are well established (**Table 1**) (133, 136, 165, 166). It was suggested that miR-150-5p should be considered a promising target for DMCP treatment since its knockdown reverses cardiac remodeling (23, 137).

## 4 lncRNAs RNA and DCM

### 4.1 General characteristics on lncRNAs

lncRNAs are heterogenous RNA transcripts with more than 200 nucleotides that are not translated into proteins (167) but can interact with DNA, RNA and proteins *via* base pairing or chemical interactions, thus exhibiting more versatile roles compared to miRNAs. RNA polymerase II transcribes lncRNAs from exonic, intergenic, or distal protein-coding regions of the genome into pre-mature lncRNAs that are polyadenylated at the 3’-end and capped on the 5’-end with methyl-guanosine (168). The precursor lncRNA undergoes alternative splicing either by interacting with specific splicing factors or forming RNA-RNA duplexes with pre-mRNA molecules (169). lncRNAs regulate gene expression at the transcriptional, translational and post-translational levels (30, 170) by binding to DNA-binding proteins (171), recruiting epigenetic complexes during DNA methylation (172), and serving as precursors of miRNAs (173). Their function depends on the cellular location; lncRNAs expressed in the nucleus regulate gene expression *via* recruitment of transcription factors or epigenetic complexes (174) whereas cytoplasmic lncRNAs participate in modulation of the mRNA stability and translation and post-translational modifications (175–177). lncRNAs are further classified as signal, guide, decoy, and scaffold lncRNAs depending on their cellular function. Signal lncRNAs respond to specific stimuli at distinct subcellular locations whereas guide lncRNAs direct ribonucleoprotein complexes to specific targets (33). Decoy lncRNAs bind and sequester regulatory proteins such as transcription factors (178), while scaffold lncRNAs have a structural role in chromatin organization as platforms for assembling ribonucleoprotein complexes (179). It has been estimated that the human genome contains over 16000 lncRNAs (Gencode-Human Release 27, https://www.gencodegenes.org/human/ *)* (180). However, despite this remarkable number, the number of functional lncRNAs remains questionable, although they express valuable cellular properties (168, 181).

### 4.2 Roles of lncRNAs in diabetic cardiomyopathy

Although there are fewer reports in the literature regarding lncRNAs’ connection to DCMP compared to miRNAs, recent evidence strongly supports the equally important emerging role of lncRNAs in DCMP pathophysiology (**Figure 1**). Levels of several lncRNAs are perturbed in serum and myocardial biopsy of patients with DCMP (182, 183). For instance, the plasma level of the lncRNA, the steroid receptor RNA activator (SRA), is decreased in DM patients with CVDcompared to DM patients without any associated complications and healthy subjects. Furthermore, a 5-year follow-up study demonstrated that perturbed levels of SRA correlate with an increased incidence of cardiovascular disease in DM patients (184). Accumulating evidence shows that lncRNAs participate in the modulation of multiple pathways associated with OS and inflammation, which are implicated as important factors in DCMP development and progression, myocardial injury, cardiac hypertrophy, and diabetic vascular complications (142). HOX transcript antisense RNA (HOTAIR) has a crucial role in the CVD pathophysiology (185), and its expression is significantly downregulated in myocardial tissues and serum of patients with DCMP compared to DM patients and healthy controls (146). HOTAIR expression was also decreased in the hearts of streptozotocin-treated mice, whereas its overexpression decreased OS and inflammation and improved cardiac function (147). HOTAIR was reported to serve as a molecular sponge of miR‐34a, which targets *Sirt1* (**Table 1**) (147, 186). HOTAIR was also shown to ameliorate DCMP by increasing the viability of cardiomyocytes *via* PI3K/Akt pathway activation (146).

In a study by Yu et al. (145), differentially expressed lncRNAs during cardiomyocytes’ OS and apoptosis induced by high glucose were identified by RNA sequencing. Consequent functional studies showed that inhibition of lncRNA NONRATT007560.2 reduces ROS generation and apoptosis, suggesting its important role in developing cardiomyopathy. In addition, it was observed that NON-RATT007560.2 have binding sites for miR-208a (145), which was previously associated with the perturbed cardiac remodeling in the myocardium of DMT2 patients (144). Xu et al. found that NONRATT021972 siRNA treatment of DM rats decreased the elevated TNF-α expression and abolished serine phosphorylation of IRS-1 in superior cervical ganglion cells, whereas downregulation of NONRATT021972 restored decreased heart rate variability in diabetic rats (**Table 1**) (143).

Another lncRNA, KCNQ1OT1, whose expression is increased in the serum of diabetic patients, as well as in high glucose-induced cardiomyocytes *in vitro*, and cardiac tissue of T1DM streptozotocin-induced diabetic mice, has been associated with pathophysiological mechanisms leading to cardiac dysfunction (138, 139, 183). A study by Coto et al. revealed that the increased levels of KCNQ1OT1 induce TGF-β1, p-Smad2 and p-Smad3 expression and are accompanied by collagen deposition, activation of fibrotic formation and cardiac remodeling, ultimately resulting in deterioration of LV function. The inhibition of KCNQ1OT1 expression significantly ameliorated cardiac function and reduced remodeling *via* TGF-β1/Smads pathway (138). Another study showed that KCNQ1OT1 silencing improves cardiac function by decreasing apoptosis *via* targeting miR-214-3p and caspase-1 gene, which leads to reduced cell death and abnormalities in cytoskeletal structure as decreased calcium overload (**Table 1**) (183).

lncRNA H19 also regulated cardiomyocyte apoptosis in diabetic cardiomyopathy (134). Li et al. reported that expression of H19 was significantly downregulated in the myocardium of diabetic rats, whereas its overexpression reduced OS, inflammation and apoptosis, leading to an improvement of LV function (134). In cultured cardiomyocytes transfected with H19 siRNA, decreased expression of H19-derived miR-675 was observed. *VDAC*1 gene, involved in cardiomyocyte apoptosis and the progression of cardiac muscle dysfunction, was identified as a target of H19/miR-675-mediated downregulation (134). Another study reported that overexpression of H19 epigenetically silences *DIRAS3* (DIRAS Family GTPase 3), promotes mTOR (mammalian target of rapamycin) phosphorylation, and inhibits autophagy in cardiomyocytes exposed to high glucose (**Table 1**) (140).

lncRNAs are also implicated in cardiomyocyte injury *via* activation of NF-κB and TNF signaling pathways. In obesity, DM and other metabolic disorders, excessive amounts of saturated fatty acids, such as palmitic acid (PA), may be deposited in cardiomyocytes causing lipotoxic damage (2, 187). Upregulation of inflammatory factors TNFα and IL-1β and lncRNA metastasis-associated lung adenocarcinoma transcript 1 (MALAT1), which plays a crucial role in cardiomyocytes ischemia-reperfusion damage, was shown in PA-treated cardiomyocytes (82). MALAT1 knockdown increased the viability of PA-treated cardiomyocytes and reduced TNF-α, IL-1β, myocardial damage markers such as lactate dehydrogenase (LDH) and CK-MB, and apoptosis (142). MALAT1 specifically binds to miR-26a, inhibiting the inflammatory signaling pathway Toll-like receptor 4 (TLR4)/NF-κB by binding to its target gene, *HMGB1*. Thus, MALAT1 inhibition alleviates lipotoxic myocardial injury *via* the miR-26a/HMGB1/TLR4/NF-κB axis (142). Downregulation of MALAT-1 also reduces inflammation under high glucose conditions. A study by Puthanveetil et al. reports significant upregulation of MALAT1 in endothelial cells exposed to high glucose levels (141). Increased MALAT1 levels were associated with a parallel increase in TNF-α, interleukin 6 (IL-6) and serum amyloid antigen 3 (SAA3), an inflammatory ligand and target of MALAT1. These findings suggest that MALAT1 regulates glucose-induced upregulation of inflammatory mediators IL-6 and TNF-α by activating SAA3 (141).

The level of lncRNA Antisense Non-coding RNA in the INK4 Locus (ANRIL) is increased in peripheral venous blood from DMT2 patients with acute myocardial infarction (188). ANRIL was shown to regulate the expression of *HBEGF* and *CDH5* genes involved in vascular permeability, leukocyte migration, and associated inflammation (148). ANRIL level is increased in the hearts of diabetic rats, and its silencing is associated with reduced levels of LDH, CK-MB, and inflammatory cytokines TNFα, IL-6, and IL-1β, suggesting that ANRIL inhibition improves cardiac function (**Table 1**) (149).

## 5 Therapeutic applications of lncRNAs

**Figure 2:**
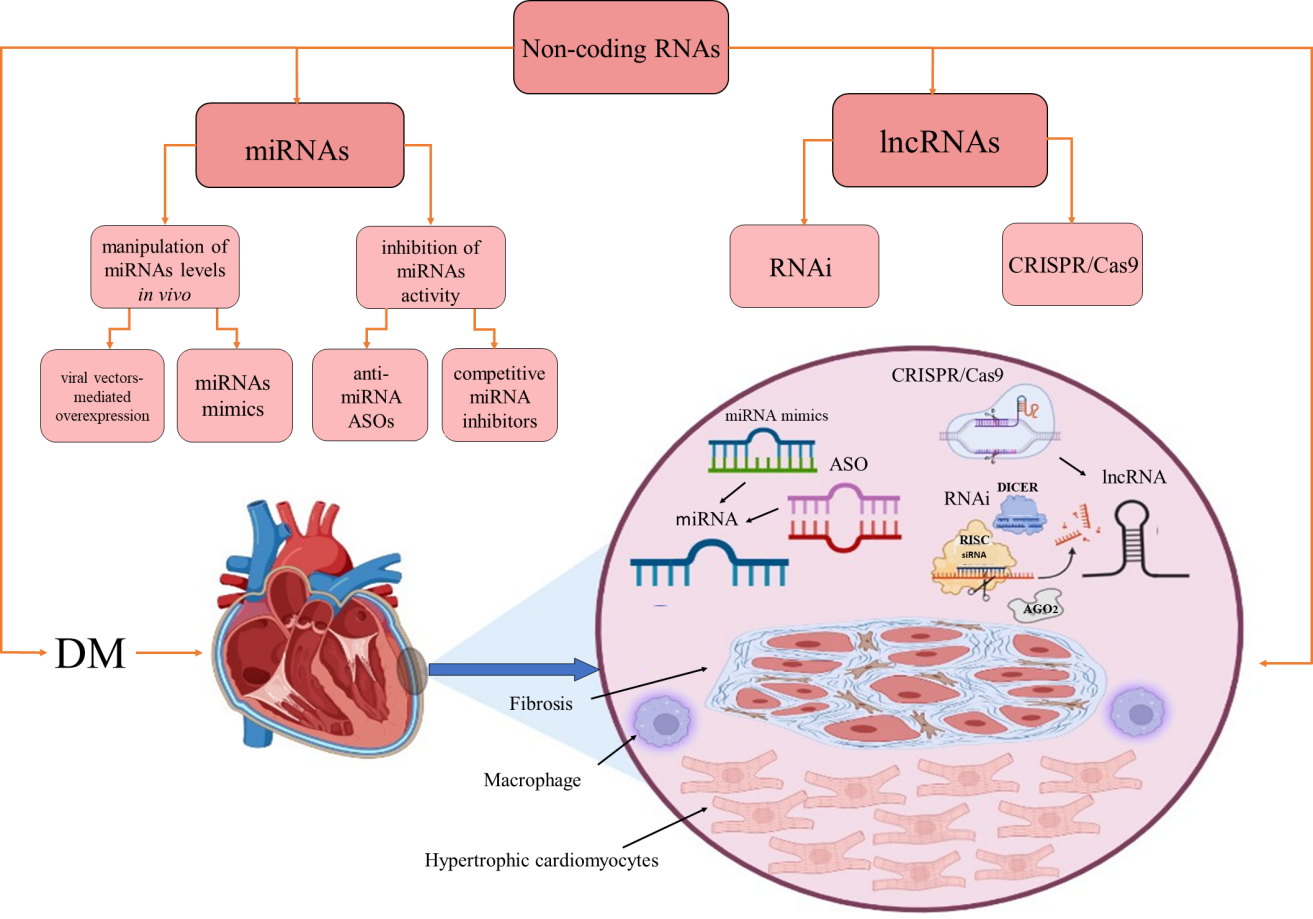
Therapeutic approaches based on miRNAs and lncRNAs. Non-coding RNAs as therapeutics in diabetes-induced cardiomyopathy. AGO2, Argonaute RISC Catalytic Component 2; ASO, antisense oligonucleotide; DM, diabetes mellitus; RISC, RNA-induced silencing complex; RNAi, RNA interference. Created with BioRender.com.

RNA-based therapies offer several significant advantages compared to other types of treatments: they allow simultaneous targeting of multiple protein-coding genes, restoration of homeostasis by fine-tuning of ncRNAs expression to their physiological concentrations, targeting of genes that are inaccessible to other therapeutic, and circumvention of drug resistance (189, 190). Manipulation of miRNA levels *in vivo* is achieved by two main strategies: restoration of downregulated miRNA levels by synthetic double-stranded miRNAs molecules called miRNA mimics or viral vectors expressing miRNA; inhibition of miRNAs activity by anti-miRNA antisense oligonucleotides (ASOs, antimiRs) or competitive miRNA inhibitors (**Figure 2**). miRNA mimics have the same sequence as an endogenous miRNA and may simultaneously target multiple mRNAs (191). So far, two miRNA mimics, miR-34 mimic MRX34 (192, 193) and the miR-16 mimic MesomiR-1 (194), have been tested in clinical trials for potential cancer treatment. Interestingly, as previously mentioned, miR-34 and mir-16 have been implicated in DCMP physiopathology.

ASOs are single-stranded DNA molecules entirely complementary to one specific target mRNA and may act by arresting protein translation *via* steric hindrance, causing RNase H-mediated mRNA degradation or altering pre-mRNA splicing by interfering with *cis*-splicing (195–197). AntimiRs are ASOs with full or partial complementarity to an endogenous miRNA that prevents its interaction with the target genes. When antimiRs are conjugated to cholesterol for improved intracellular delivery, they are called antagomiRs (198). Two miR-122 antimiRs, miravirsen (SPC3649; β-D-oxy-LNA) and RG-101 (*N*-acetylgalactosamine-conjugated ASO), have been clinically tested in the context of the development of potential hepatitis C virus therapeutics (199). Anti-miR-92a (MRG-110) was clinically tested for its ability to promote angiogenesis and improve wound healing (197). It should be mentioned that the instability of RNA therapeutics, combined with their inability to cross cell membranes due to their negative charge, required various chemical modifications to improve their pharmacokinetics and pharmacodynamics properties (197, 200, 201). First-generation modifications improved stability by replacing phosphodiester with phosphorothioate (PT) backbone linkages. Second-generation modifications improved bioavailability while reducing toxicity and immunostimulation by replacement of the 2′-*O*-alkyl group of the sugar moieties with 2′-O-Me, 2′-MOE or 2′-F. Third-generation modifications are based on modifications of the furanose ring to create peptide nucleic acids (PNAs), locked nucleic acids (LNAs), and phosphoramidite morpholino oligomers (PMOs). All currently approved RNA therapeutics for clinical investigations have second or third-generation chemical modification (197).

Several antimiRs were tested in experimental animal models in the specific context of DCM. For instance, the administration of antagomiR-155 decreased cardiac infiltration of inflammatory mediators and ameliorated myocardial damage and overall cardiac function (202). However, it was observed that estrogen deficiency in DCM mice increased inflammation due to the excessive infiltration by pro-inflammatory M1 macrophages (203). Estrogen-dependent DCM aggravation was successfully prevented by treatment antagomiR-155 conjugated to gold nanoparticles, improving the heart’s structure and function. It was suggested that a therapeutic approach based on miR-155 inhibition might serve as a promising strategy for ameliorating cardiac function in DCM (203). Also, in the post-infarcted heart of a preclinical animal model, it was shown that an intracoronary injection of antagomiR-92 encapsulated in poly(lactic-co-glycolic acid) stimulated angiogenesis and improved myocardial function (204).

lncRNA-targeting therapeutics have recently become the focus of investigations, but so far, no such therapeutic has entered clinical trials. lncRNAs are currently extensively studied as clinical biomarkers for various diseases, but it could be envisioned that they may serve as novel targets for RNA interference (RNAi) and CRISPR/Cas9 gene-editing interventions. RNAi approach is based on the use of exogenous double-stranded small interfering RNA for specific knockdown of target RNAs by engaging a degradation pathway that involves DICER, a multiprotein RNA induced silencing complex (RISC) and the endonuclease AGO2 (205). Several lncRNAs were successfully knocked down using the RNAi *in vitro*. However, their silencing *in vivo* remains challenging, partly due to the lack of efficient delivery methods (190, 206). Clustered Regularly Interspaced Short Palindromic Repeats/associated protein-9 nuclease (CRISPR/Cas9) can be used for editing the whole human genome, including ncRNAs. CRISPR/Cas9 RNA-guided editing platform consists of a Cas9 nuclease that binds to a conserved sequence consisting of three nucleotides, called proto-adjacent motif (PAM), and a short CRISPR RNA (crRNA) that acts as a guide for Cas9, together with an adaptor trans-activating RNA (tracrRNA). The crRNA and tracrRNA can be fused to create the single-guide RNA that can direct Cas9 to any target in the proximity of the PAM sequence (207–209) and create a double-stranded DNA break. The CRISPR/Cas9 platform was used to target the expression of miRNAs implied in various pathophysiological conditions (73, 210, 211), but it can also be employed for lncRNA overexpression or transcriptional repression. CRISPR/Cas9 platform has been used for high-throughput profiling of lncRNAs associated with pathophysiological conditions, especially in oncology (212). Successful use of this editing platform for lncRNA manipulations may require targeting the lncRNA splice acceptor/donor sites (157, 213), precise delivery of CRISPR to specific tissues, and improved control of its off-target effects (214).

## Conclusions

miRNA and lncRNA deregulation, in addition to their association with systemic and organ-specific inflammation (via interactions with PPAR and Nrf2, as well as PI3KT/Akt and NF-κB), qualify them as important DCMP diagnostic and treatment tools. Future extensive research must identify miRNAs and lncRNAs as biomarkers and therapeutic targets shared by different components of the metabolic disease cluster.

## Author contributions

MM - wrote the article. ZG - wrote the article, JR - wrote the article, ME - wrote the article, XG- wrote the article, and EI - wrote and critically reviewed the article. All authors contributed to the article and approved the submitted version.
